# Breakthrough Assembly of a Silk Fibroin Composite for Application in Resistive Pressure Sensing

**DOI:** 10.1021/acsapm.5c00242

**Published:** 2025-04-10

**Authors:** Giuseppe De Giorgio, Valentina Vit, Davide Vurro, Benedetta Guagnini, Bianca Zumbo, Nicola Coppedè, Gianluca Turco, Giuseppe Tarabella, Pasquale D’Angelo

**Affiliations:** 1 Institute of Materials for Electronics and Magnetism (IMEM), National Research Council (CNR), P.co Area delle Scienze 37/A, Parma 43124, Italy; 2 Department of Medicine, Surgery and Health Sciences, University of Trieste, Piazza dell’Ospitale 1, Trieste 34129, Italy

**Keywords:** silk fibroin, PEDOT:PSS, pressure sensors, biomaterials, electronics, conductive sponges, freeze foaming, green chemistry

## Abstract

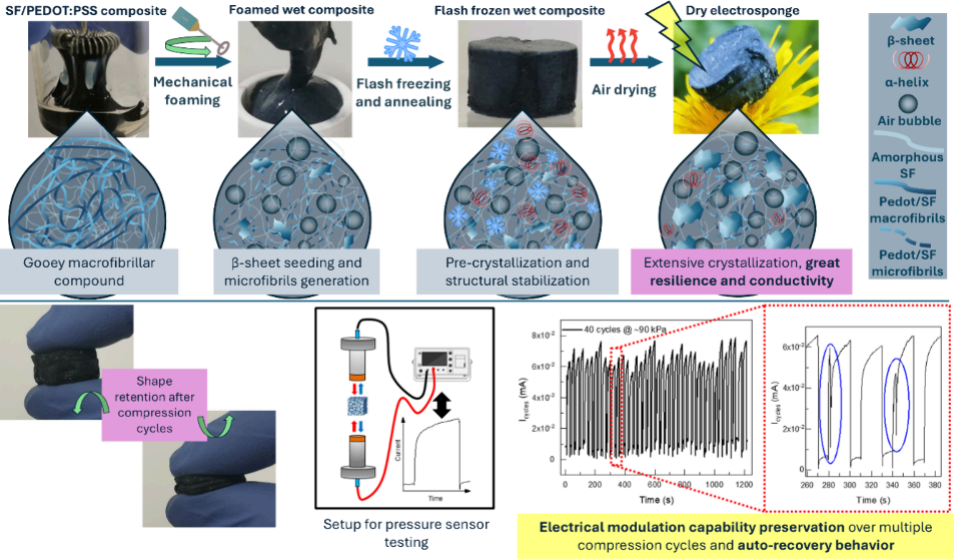

Driven by the dictates of sustainability, we have designed, realized, and optimized a method for easy development of biocompatible, highly porous, and electrically conductive 3D structures from the combination of natural and synthetic polymers for pressure sensing applications. In particular, a foaming method followed by a fast freezing step, both performed on blends made of silk fibroin (SF) aqueous solution, PEDOT:PSS electrically conductive polymer, and water-soluble PVA, has allowed the fabrication of conductive electrosponges, intrinsically integrating the structural and electrical counterparts of a resistive pressure sensor in a single “green” material. An exhaustive analysis of their structural (with FTIR), morphological (with μ-CT), and mechanical (by means of stress–strain measurements) properties has been performed, of which the latter was coupled with the electrical characterization of the electrosponges while undergoing compression–decompression cycles. PVA addition has been recognized as crucial for conferring to the material the right compromise among elasticity, recovery attitude, and resilience/durability to the proposed constructs. The fabricated electrosponges show a promising combination of mechanical and electrical properties, with the former induced by both the highly porous structure of the foamed/frozen compound and the elasticity enhancement induced by PVA, whose concentration influences the electrosponge resilience and recovery attitude. Based on the results from the material characterization, the composite with 1% v/v PVA content has shown the best compromise among elasticity, resilience, and shape recovery. The related sensor shows a sensitivity comparable to other hybrid SF composites (10^–3^ kPa/mA vs 10^–3^–10^–2^ kPa/decade), an applied stress magnitude-dependent swiftness (from hundreds of milliseconds to few seconds), and an exhaustive current recovery on numerous repeated compression–decompression cycles in wet conditions.

## Introduction

The engineering of synthetic and natural materials may favor pressure sensing in exploiting the monolithic integration of sensing and transduction elements in a bifunctional conducting/elastic material, which is able to implement an electronic transduction of mechanical pressure inputs on it. Pressure sensors have an established application in industry^1^ and the automotive sector,^2^ while recent groundbreaking applications have gained attention in various fields, such as robotics,^3^ health monitoring,^4−7^ and even human–computer interaction^8,9^ and textile electronics.^10^ Most sensors are made of petroleum-based polymers from nonrenewable sources and produced through environmentally unfriendly processes.^11^ Numbers provided by the literature indicate that an estimation of the amount of plastics produced all over the world per year is extremely complicated. Conversely, the need to prevent the strong environmental impact of the production and wasting of plastic materials, known to be harmful to human health, has captured today’s research in materials science. In this light, the use of natural protein-based biopolymers could replace synthetic polymers by mimicking their structure and function while offering several advantages such as complete degradation, reduced waste management, biocompatibility, implantability, and, importantly, the as-needed customization of their chemical–physical properties through easy chemical engineering approaches.^12^ In particular, fibrous proteins, which are biopolymers composed of a primary sequence of standard amino acids, confer structure and function to several biological systems. Hence, they correspond to natural building blocks. In this context, one of the most studied and characterized protein-based biopolymers is silk fibroin (SF), in particular the one derived from the silk of *Bombyx mori* (silkworm) cocoons, due to the high yield of the related extraction process.

The primary amino acid sequence of a protein dictates its folding into secondary structures. The SF primary structure is characterized by a highly redundant amino acid composition that, when folded, organizes itself into packed β-sheet hydrophobic domains (β-crystallites), interspersed with hydrophilic regions and generating ordered structures. Thus, folded SF generates a natural block copolymer with a high degree of crystallization and extraordinary properties, i.e., a combination of mechanical strength, toughness, and elasticity, but also low immunogenicity and biodegradability.^13,14^ SF is extracted from raw silk through the degumming process where sericin, the other major constituent of silk, is removed, obtaining degummed SF fibers.^15^ Degummed SF is then dissolved using different approaches in order to obtain an aqueous SF solution that can be regenerated by adopting several techniques, producing silk-based systems with different mechanical and chemical–physical properties, such as 3D porous structures, films, hydrogels, and nanoparticles.^16,17^ It is worth noting that SF crystallization represents the key for imparting to a given fibroin-based material a long-lasting and desired shape, whereas an extensive β-sheet generation often becomes a critical point for SF regeneration. Usually, SF crystallization makes use of chemicals as alcohols that, on sequestering water from the amorphous SF aqueous solution, increase the protein chain–chain contact, triggering the regeneration of β-sheet structures and natural recrystallization.

Most literature methods for SF regeneration are aimed at promoting the assembly of SF-based highly porous 3D materials. Among these, freeze-drying, salt leaching, and gas foaming^18^ are the most common and used approaches. SF gas foaming, for instance, has been used to produce SF foams suitable for *in situ* injection of a porous bioactive degradable filler aimed at supporting tissue regeneration.^19^ Moreover, supercritical foaming of polylactide (PLA) composites has been demonstrated to generate interesting 3D structured scaffolds to meet various requirements in tissue engineering and green chemistry applications.^20^ On the other hand, although widely employed,^21^ freeze-drying and salt leaching show some disadvantages owing to the difficulty of controlling some parameters of the structures being manufactured with, such as the porosity and homogeneity of the material.^18^

Poly(3,4-ethylenedioxythiophene):poly(styrenesulfonic acid) (PEDOT:PSS) represents one of the most valuable and successful electrically conductive polymers, with excellent electrical conductivity, biocompatibility, water processability, and flexibility.^22−24^ PEDOT:PSS can be combined with SF to confer electrical properties to related composite materials while ensuring them to preserve the biopolymer’s mechanical properties. Of course, the provided conduction offers potential applications of SF composites in electronics and biomedicine.^25,26^

As it concerns the world of sensors, SF has been largely used to fabricate an innovative generation of biopolymer-based strain and pressure sensors.^5,11,27^ In this respect, the use of dopants in fibroin represents an approach to realizing conducting fibroin composites, thus enabling solutions for the design and implementation of piezoresistive sensors. For instance, carbon nanotubes have been dispersed into a fibroin hydrogel to realize a conducting material with a promising elastic modulus (from units to hundreds of kPa) and stretchability to be used as wearable pressure and strain sensors,^28^ while graphene oxide mixed with a PEDOT:PSS solution has allowed to fabricate a composite hydrogel made of SF, polyacrylamide, PEDOT:PSS, and graphene oxide, with similar properties.^29^ In addition, Xu et al. assembled conductive microspheres using SF/PLGA (poly(lactic-*co*-glycolic acid)) fibers as an inert support for SiO_2_/polyaniline (PANI) microspheres. The resulting material is flexible and has fast response time (145 ms) and good durability (over 2000 cycles) when used as a piezoresistive pressure sensor.^30^ Another interesting example based on a different transduction mechanism has been shown by Li et al. In this case, a SF hydrogel has been synthesized trying to mimic human epidermis–dermis structure. A capacitive pressure sensor based on three layers of SF-glycerol films and FS hydrogel has been shown in this case.^31^

In line with the as-reported examples, most literature works pertain the use of fibroin as an elastic and flexible support to implement the sensing element, while the transduction is performed by conducting networks coupled with or embedded in a biopolymeric matrix or even by exploiting the dielectric properties of the biopolymer integrated into a capacitor device architecture.

Technologically, the structural integration of sensing and transduction elements in a raw material may be desirable in view of easy device fabrication, even by additive manufacturing routes, and in-line process capability for monolithic integration of sensors in daily life objects.

Herein, we propose an innovative and simple foaming methodology avoiding the use of tricky synthesis routes and chemicals (e.g., alcohols, cross-linkers) to implement a sponge-like conducting biocomposite showing elastic properties suited to the realization of solution-processable integrated pressure sensors, responsive in a wide range of applied pressures. Specifically, our method is based on mechanical foaming of the aqueous SF blended with PEDOT:PSS, with the addition of PVA (1% and, by comparison, 0.5% v/v) acting as an elasticity enhancer. The foamed blend was sequentially flash-frozen in liquid nitrogen in order to stabilize the bubbled structure, allowing the preservation of a 3D network, annealed at −20 °C overnight (O/N) to allow the SF crystallization through the formation of β-sheets and β-crystallites, and, finally, air-dried at 60 °C. As demonstrated by μ-CT, SEM, and FTIR analysis, the supramolecular interaction between PEDOT:PSS and SF was enhanced using this method and PVA has shown to ameliorate the elasticity of the system after air drying. Finally, a sustainable, biocompatible, and highly conductive 3D porous material, acting as an electrosponge, was obtained. SF/PEDOT:PSS/PVA electrosponges demonstrated great mechanical properties, resilience, and high sensitivity in the electrical responsiveness on compression. As a case study, the SF/PEDOT:PSS/PVA electrosponge was optimized in terms of PVA content for better mechanoelectrical properties and applied for the development of pressure sensors. The proposed manufacturing method is highly reproducible, even on a large scale and permits fabrication of 3D objects of different shapes and sizes with potential applications in many fields, not only in pressure sensing.

## Experimental Section

### Materials

Lithium bromide (LiBr) was purchased from Alfa Aesar (Haverhill, United States). Sodium carbonate (Na_2_CO_3_) and poly(vinyl alcohol) (PVA, m.w. 115,000) were purchased from VWR (Carnaxide, Portugal). PEDOT:PSS (Clevios PH 1000) was purchased from Heraeus (Leverkusen, Germany). Ethylene glycol was purchased from Sigma-Aldrich (Saint Louis, United States).

### SF Preparation

SF aqueous solution was obtained from *Bombyx mori* cocoons by boiling in 0.02 M Na_2_CO_3_ solution for 20 min and thoroughly rinsing in Milli-Q water to completely remove the wax and gluey sericin residues. Washed SF fibers were allowed to dry overnight and dissolved in 9.3 M LiBr solution at 60 °C, and after complete dissolution of the fibers, the SF solution was dialyzed for 3 days against ultrapure water using dialysis membranes with a molecular weight cutoff of 10 kDa and a flat width of 45 mm (Spectra/Por 6, VWR, USA). After the dialysis step, the final aqueous SF solution was centrifuged at 5000 rpm for 20 min two times to remove impurities and unsolved portions.

### Fabrication of SF/PEDOT:PSS and SF-PEDOT:PSS/PVA Electrosponges

For the fabrication of electrosponges, the aqueous SF solution was concentrated by gently stirring at 60 °C on a hot stir plate. The obtainment of a 10 wt % working concentration was confirmed by drying a sample of the solution at 60 °C for 4 h and weighing the remaining solid. Meanwhile, PEDOT:PSS was sonicated for 30 min at 35 Hz to break down large aggregates. The solution was then filtered through a 0.45 μm syringe filter. Subsequently, ethylene glycol was added at a concentration of 5% v/v to enhance the conductivity of PEDOT:PSS. For the analysis of the primary viscous, gooey composite, SF and PEDOT:PSS were mixed in equal parts (1:1). A sample from this initial mixture was taken and dried at 60 °C for 4 h prior to SEM investigation. To prepare SF/PEDOT:PSS electrosponges, equal parts of the two components were mixed and vigorously whipped for 1 min at 11,000 rpm using a commercial milk frother. This process allowed air incorporation and the disruption of macrofibrils. The foamy compound was cast into cylindrical molds (2 cm diameter, 1 cm height) and flash-frozen by dipping the molds into liquid nitrogen for 2 min. The primary freezing step was followed by an annealing phase at −20 °C overnight. Subsequently, the annealed sponges were extracted from the molds and dried at 60 °C for 4 h. To enhance the resilience and mechanical properties of the electrosponges, alternative formulations were fabricated by adding 0.5% or 1% PVA. Separately, a 10 wt % PVA solution was prepared by dissolving PVA flakes in ultrapure water. The solution was stirred for 6 h at 80 °C to ensure complete dissolution and then stored at 4 °C. PVA, at a final concentration of 0.5% or 1 wt %, was added to the SF/PEDOT:PSS 1:1 composite prior to the foaming step. The synthesis then proceeded as described above. For the preparation of samples composed only by SF, the 10 wt % working solution was foamed and elaborated following the same procedure without the addition of other components.

The synthesized SF/PEDOT:PSS/PVA composites were cast into molds of 2 cm diameter and 1 cm high. The actual diameter after the drying process for electrosponge synthesis was 1.1 cm, while the height of 1 cm was preserved.

### FTIR-ATR Analysis

To obtain FTIR spectra, the samples examined were SF sponge (without other components), SF/PEDOT electrosponge, and SF/PEDOT/PVA with 1% PVA electrosponge. Dry samples were analyzed by infrared spectroscopy in attenuated total internal reflection mode (ATR) (Cary 630, Agilent), and total spectra were recorded in a range of 4000–700 cm^–1^. For the deconvolution, the spectral range from 1700 to 1450 cm^–1^ was selected to specifically analyze the conformational states of Amide I and II, and respective spectra were deconvoluted by using Origin Pro 2018 software (OriginLab Corporation).

### Morphological Analysis by μCT

μCT of samples was performed by means of a cone-beam system called TOMOLAB (www.elettra.trieste.it/Labs/TOMOLAB).^32^ The device is equipped with a sealed microfocus X-ray tube, which guarantees a focal spot size of 5 μm in an energy range from 40 up to 130 kV, and a maximum current of 300 μA. A CCD digital camera was used with a 49.9 × 33.2 mm^2^ field of view and a pixel size of 12.5 × 12.5 μm^2^. The samples were positioned onto the turn-table of the instrument, and acquisitions were performed with the following parameters: distance source–sample (FOD), 80 mm; distance source–detector (FDD), 264 mm; magnification, 4×; hardware binning, 2 × 2; resolution, 8 μm; tomographies dimensions (pixels), 1984 × 1024; slice dimensions (pixels), 1984 × 1984; number of tomographies, 1440; number of slices, 864; *E* = 90 kVp, *I* = 55 μA; exposure time 1.5 s. The slice reconstruction process achieved by means of commercial software (Cobra Exxim) started once the tomographic scan was completed, and all the projections were transferred to the workstation. Input projections and output slices were represented by files (one file per projection and one file per slice) using arrays of 16-bit integers. Three-dimensional μCT image analysis was performed using Fiji software.

### Mechanical Characterization by Stress–Strain Measurements

Mechanical evaluation of the elastic modulus was performed on two control sponge samples made up of only 2.5% and 5% SF and on the SF/PEDOT:PSS/PVA (1% w/v) sample by means of a Galdabini SUN 500 (Galdabini) Universal Testing Device (Galdabini, Cardano al Campo, VA, Italy) with a crosshead speed of 1 mm/min. Force (N) and crosshead travel (mm) recorded by the universal testing machine were then converted to stress and strain. Samples were compressed up to a force limit of 80 N with a cylindrical pressor (Ø = 2 mm).

Resistivity behavior of the materials under compression was performed on gold-sputtered and non-gold-sputtered 0.5% PVA and 1% PVA samples. To ensure the presence of a conductive surface, samples were sputter-coated with gold (Sputter Coater K550X, Emitech, Quorum Technologies Ltd., UK). Both gold-sputtered and non-gold-sputtered samples were compressed by means of a universal testing machine (AGS-X 10, Shimadzu Corporation, Kyoto, Japan) with a crosshead speed of 1 mm/min. Upper and lower sample surfaces were compressed by means of a custom-made pressor, which mainly consists of two flat metallic surfaces electrically isolated from the rest of the device by means of 3D printed PLA supports. During the compression test, the two flat metallic surfaces were connected to a Fluke Model 87 V Digital Multimeter (Everett, WA: Fluke Corporation) in order to measure the electrical resistance. This latter measure was then converted to electrical resistivity, considering constant the contact surface of the sample and the variation of the sample’s length due to compression.

### Electrical Characterization of SF/PEDOT:PSS/PVA Pressure Sensors

The response of fibroin-based SF/PEDOT:PSS/PVA electrosponges to applied pressure stimuli was tested with a homemade system interfaced with a high-resolution electrometer (B2987A, Keysight), sketched in Figure 6A. The system is made of two Teflon pistons, Ø = 1.8 cm and length 10 cm, with facing bases ending with two copper plates (Ø = 1.8 cm) acting as current collectors. The sample may be located between the copper plates of the pistons and then is pressed under the action of different weights, corresponding to applied pressures ranging from 45 to 355 kPa. In our case, current vs time measurements were made at a fixed voltage bias of 0.5 V and by applying each of the selected weights for 5 s, followed by 5 s of release before the subsequent application of a heavier weight. All the measurements were repeated in triplicate. The same measurement was performed for the PVA-free SF/PEDOT:PSS composite to address the role of PVA as an elasticity enhancer.

Compression tests were also performed on wet samples. In this case, dry samples of the 3D shaped compounds were humidified before performing the pressure tests as described above. Specifically, samples were dipped into distilled water for 5 min and then dried in air for 20 min to remove the excess water.

Cyclic tests consisting of the application of 40 cycles of a step-like pressure, step height of 90 kPa applied for 20 s, followed by 10 s of weight release, were performed on the benchmark sample, i.e., the composite containing 1% of the additive PVA.

For longer pressure stability tests, cyclic tests were performed by applying 300 cycles of a step-like pressure with 85 kPa on the sample with 1% PVA for 1 s, pausing for 2 s between each stimulus. The resultant currents were normalized and plotted.

The fibroin-based material containing 1% PVA was used to calculate the response time, τ, again applying a voltage of 0.5 V under four compression steps corresponding to a 3 min long application of pressure stimuli, ranging between 45 and 180 kPa, followed by 1 min of release for each applied weight. Saturating current vs time curves were analyzed by fitting them using a double exponential, as done by Preziosi et al.:^33^
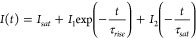
1

Here, τ_rise_ corresponds to the sensor swiftness and τ_sat_ measures the time needed to reach an ideal saturation of the current on compression. I_sat_ is the ideal saturation current, while I_1_ and I_2_ are the contributions to the current time-evolution at the starting compression and during the settlement of the electrosponge structure in response to the applied pressure, respectively.

## Results and Discussion

### SF/PEDOT:PSS and SF/PEDOT:PSS/PVA Composite-Based Electrosponge Fabrication and Macroscopical Characterization

**Figure 1:**
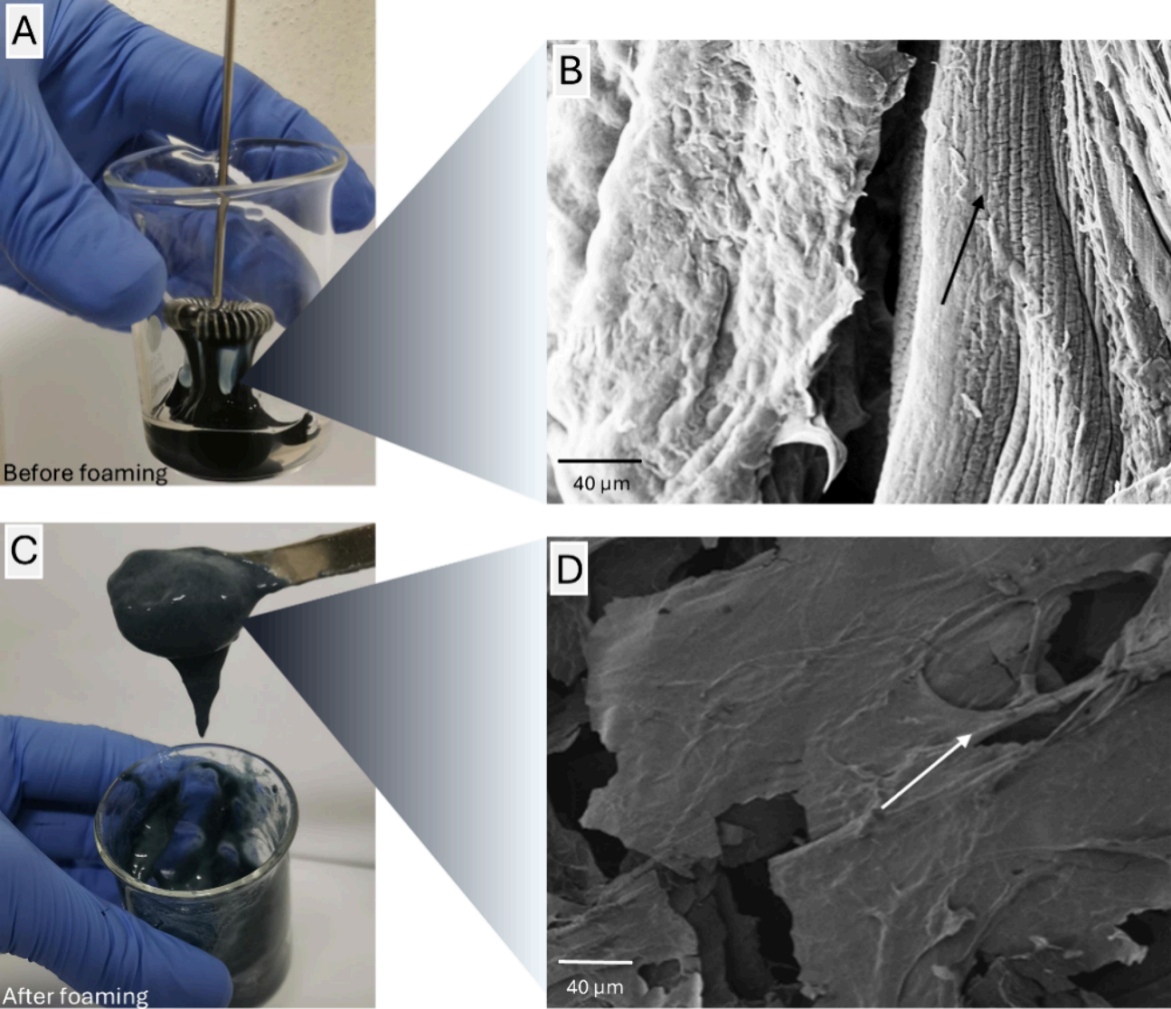
Processing of the SF/PEDOT:PSS composite. In (A), it is shown as the viscous gooey composite (before foaming) given by the primary interaction between the two phases. The primary composite is characterized by macrofibrillar structures, which are clearly visible in the SEM image (B) of a dried composite sample (black arrow). After the foaming of the composite, a dense and stable foam is obtained (C). The process causes the disruption of macrofibrils turning into microfibrils detected by SEM analysis (D, white arrow).

SF/PEDOT:PSS composite preparation was carried out by mixing equal parts of SF and PEDOT:PSS aqueous solutions. The mixing of the two biocompatible polymers initially triggers a primary interaction consisting of SF fibrillation;^26^ here, it is obtained a gooey and fibrillar composite (Figure 1A). However, the viscosity of this compound greatly limits the interaction between the two raw components, since the internal layers of fibroin fibrils are unable to fully interact with PEDOT:PSS (Figure S1) after the drying of the composite on gentle mixing. To overcome this limitation, the compound was thoroughly elaborated through mechanical foaming of the gelatinous compound. The foaming process greatly ameliorated the integration between the two main components, determining an extensive interaction between SF portion and PEDOT:PSS portion. In this light, foaming provoked the mechanical disruption of the macrofibrillar primary viscous compound, allowing the formation of a dense microfibrillar foam,stabilized by a stronger chemical and spatial interaction between the two polymers. SEM analysis reveals how the macrofibrillar nature of the initial composite guided by the fibrillar SF tendency (Figure 1B) in the foamed compound (Figure 1C) turns into a microfibrillar nature (Figure 1D), confirming a greater surface–volume interaction between the components.

**Figure 2:**
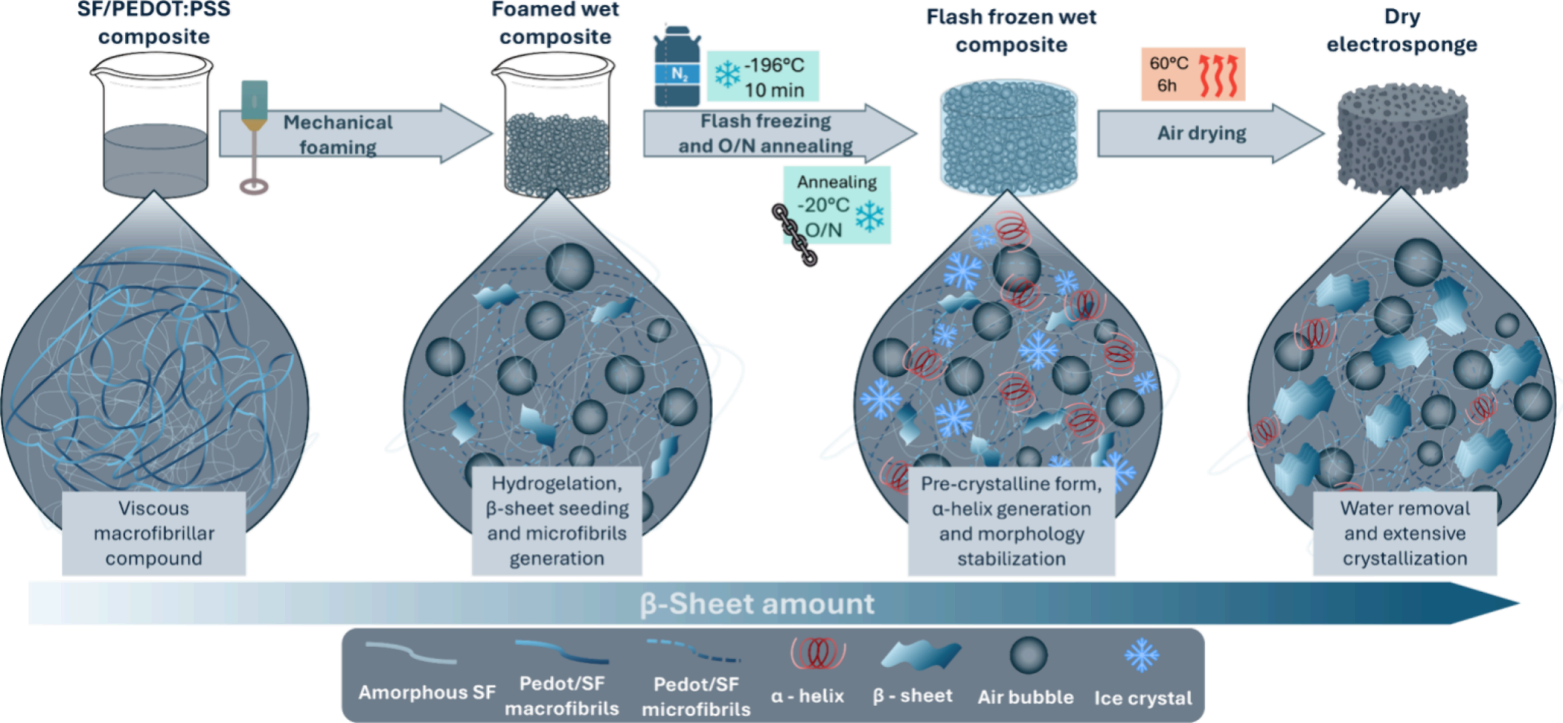
Schematic description of physical–chemical and molecular events occurring in the electrosponges obtained by the frozen foaming synthesis process.

The mechanical elaboration of aqueous SF solutions triggers the generation of β-sheets, and hydrogelation events, potentially acting as seeders for further SF extensive crystallization, can also explain the compound morphology stabilization.^34^Figure 2 reports the scheme of molecular events occurring during the described synthesis process. After the mechanical foaming, a high crystalline degree was reached following a flash freezing of the wet foams in liquid nitrogen.

The combination of the foaming technique and fast freezing curing guarantees the stabilization of SF in a precrystalline conformation, generating α-helical structures,^35^ and the maintenance of both the desired shape and the internal morphology of the material. A material with higher crystallinity degree, hence with greater mechanical properties, was obtained by an overnight (O/N) annealing step performed at −20 °C, a temperature ranging into the glass transition zone of SF in aqueous solution.^18^ Annealing of flash-frozen composites enables and stabilizes the structural arrangement of more clustered fibroin molecules afforded by ice crystal formation into the matrix. These circumstances finally trigger the self-aggregation of spatially concentrated fibroin clusters, which will naturally revert from amorphous conformations or α-helical structures to a stable β-sheet conformation simply by holding a conformational state with a lower energetic level.^36^ After the mold extraction, frozen foams were dried at 60 °C and highly porous 3D electrosponges were obtained; the final water molecule removal also allowed the dry electrosponges to reach an even higher degree of crystallization.

### SF/PEDOT:PSS and SF/PEDOT:PSS/PVA Composite-Based Electrosponge Spectroscopic Structural Characterization via FTIR

For the structural characterization of SF-based composites, the sample crystallization degree was assessed by Fourier transform infrared (FTIR) analysis. The investigation of SF secondary structure is crucial for the SF composite characterization in order to assess its mechanical performance. Amide groups present in proteins have peculiar vibrational modes, in particular C=O stretching at 1700–1590 cm^–1^ (Amide I), N–H bending and C–N bending at 1590–1460 cm^–1^ (Amide II), and C–N stretching and N–H bending at 1190–1280 cm^–1^ (Amide III). Differences in the Amide’s vibrational modes are strongly influenced by the protein's secondary structures (β-sheets, α-helix, random coils) whose presence determines frequency shifts.^37,38^ FTIR spectral analysis and deconvolution of the Amide’s peaks were exploited to get information about the structural state of SF in our samples. We focused our attention on the Amide I and II peaks.

**Figure 3:**
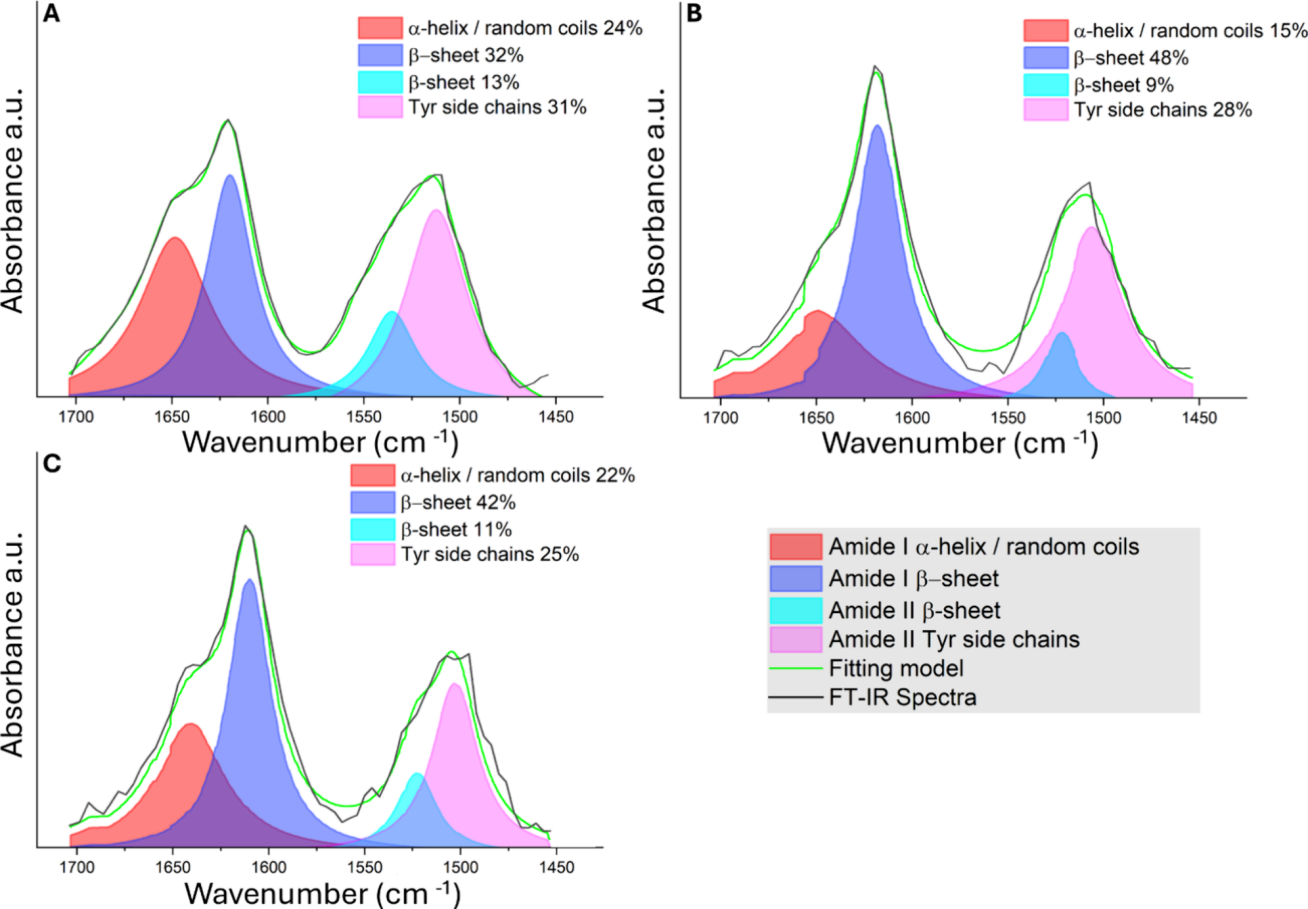
Amides I and II ATR-FTIR spectral deconvolution comparison and relative secondary structure composition percentages. SF in this case represents a control sponge made up of only SF (panel A), while SF/PEDOT (panel B) and SF/PEDOT/PVA (panel C) represent two electro-spongy composites, of which the latter has 1% v/v PVA.

FTIR analysis was performed on SF and SF/PEDOT composite formulations and are reported in Figure S2. The analysis of FTIR deconvolution spectra (Figure 3) for β-sheet monitoring in the composites underlines how the process of sponge fabrication and crystallization performed only on SF (Figure 3A), which serves as the control, reaches an important crystallization degree (total β-sheet 42%). As mentioned above, in this case, the amount of material crystallization may be triggered by mechanical foaming together with low-temperature processing and final material dehydration. Deconvolution spectral comparison between SF/PEDOT:PSS (Figure 3B) compound and SF control sample displays how the addition of PEDOT:PSS enhances crystallization of the material (total β-sheet 57%), possibly by chemical interactions between the two components that further enhanced β-sheet generation.

Starting from the as-prepared SF/PEDOT:PSS compound, a composite formulation with the addition of PVA was prepared in order to enhance the toughness and elasticity of electrosponges in view of their practical application in pressure sensing. 1% v/v PVA addition to the SF/PEDOT:PSS composite slightly lowers the β-sheet content (total β-sheet 53%) by increasing the percentage of water retained by the material due to PVA, as shown in the FTIR spectrum (Figure 3C). Additionally, PVA might have a limiting effect for β-sheet development thanks to the strong bonding that it establishes with SF through hydroxyl and carboxylic groups.^39^ This effect is enhanced by PVA that additionally maintains water molecules close to the SF primary chain, thus sterically reducing extensive material recrystallization. In addition, a PVA network formation exerting a plasticizing role in SF composites when subjected to high stress conditions could represent a crucial point for sponges with higher resilience and shape preservation.

### Mechanical Characterization of SF and SF/PEDOT:PSS/PVA Electrosponges via Stress–Strain Analysis

**Figure 4:**
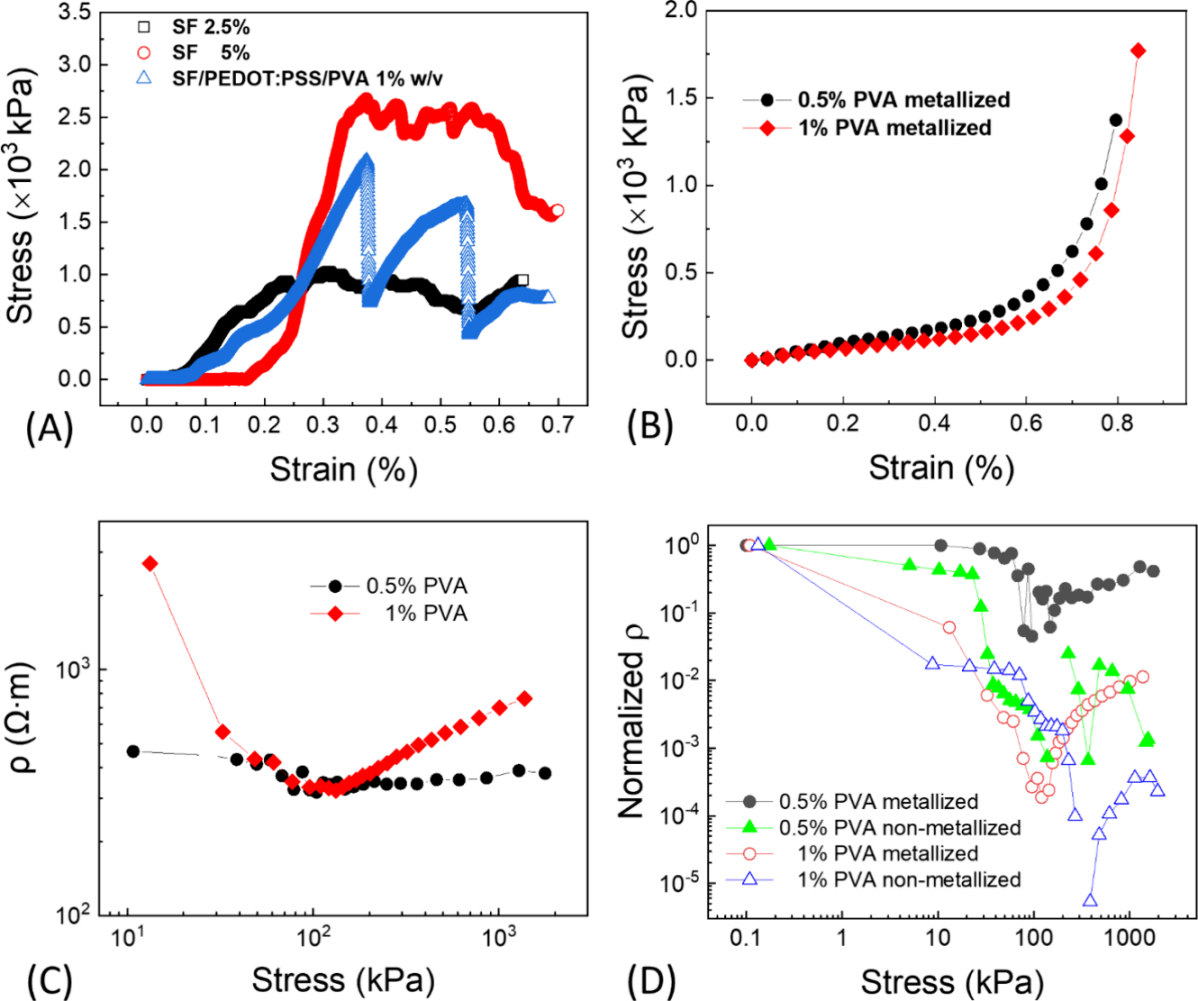
(A) Stress–strain measurements (intender Ø = 2 mm) for foamed SF samples from fibroin water solutions at 2.5% and 5% biopolymer contents and for the SF/PEDOT:PSS/PVA (1% w/v) electrosponge. (B) Stress–strain curves of SF/PEDOT:PSS/PVA 0.5% (black symbols-line) and SF/PEDOT:PSS/PVA 1% (red symbols-line) electrosponges (stress applied over the whole sample surface, Ø = 11 mm), performed on metallizing the electrosponge opposite surfaces and using conducting plates for resistivity vs compression measurements. (C) Resistivity vs stress for samples of panel B. (D) Normalized resistivity vs stress for metallized and nonmetallized SF/PEDOT:PSS/PVA, 0.5% and 1% v/v.

The mechanical properties of dry sponges fabricated using only SF, with two different biopolymer concentrations of 2.5% and 5% w/v, or by producing a 5% SF composite with 1:1 PEDOT:PSS and 1% v/v PVA (SF/PEDOT:PSS/PVA) were evaluated by the stress–strain curves, reported in Figure 4A. Stress–strain measurements were performed using an indenter, 2 mm in diameter, on cylindrical samples (Ø = 11 mm, *h* = 10 mm) to evaluate the local effects of compression on the samples under study.

The 2.5% fibroin-based sample shows enhanced elastic behavior in the low-strain region with respect to the 5% one. An increase in fibroin water content promotes, in fact, a higher sponge porosity responsible for an enhancement of the elastic modulus of the 3D spongy sample, namely, from 5.3 to 20.6 MPa. An elastic behavior is however found for increasing applied stresses, up to around 10^3^ kPa for the 2.5% fibroin sample and exceeding 2 × 10^3^ kPa for the 5% fibroin-based one. Before that, a plastic behavior takes place in both samples. From this analysis, both the softness of the 2.5% foamed fibroin sample, due to high porosity, and the robustness of the structure of porous samples assembled starting from 5% bare fibroin aqueous solution, may be evidenced. The latter fibroin concentration is accordingly best suited to support heavy loads.

The addition of PEDOT:PSS to SF, which is aimed at conferring electrical conduction to the foamed composite, in principle could affect the mechanical properties of foamed/fast frozen fibroin, as bare PEDOT:PSS uses to display a stiff character, with a Young’s modulus from 500 MPa^40^ to 2–3 GPa,^41^ far higher than tens of MPa.

The use of PVA to modulate the elasticity of the conducting SF/PEDOT:PSS composite provides an elastic modulus of 7 MPa; hence, it allows for the composite approaching the stiffness of the bare, low-density SF with a desirable elasticity, although the stress–strain curve of the SF/PEDOT:PSS/PVA composite shows anomalies on compression consisting of a loss and recovery of elasticity, which may be ascribed to modifications of the morpho-structure of the foamed electrosponge on compression.

Stress–strain measurements for SF/PEDOT:PSS/PVA 0.5% and SF/PEDOT:PSS/PVA 1% cylindrical samples (reported in Figure 4B) are performed on metallized sample surfaces (Ø = 11 mm), aimed at simultaneously collecting resistivity measurements on applied stress and ameliorating the distribution of compression over the whole section of the SF/PEDOT:PSS/PVA cylinder. Some slight differences for composites with different PVA content percentages may be spotted from the assessed stress–strain curves, meaning that small contents of PVA in the SF/PEDOT:PSS composites are effective in determining a sustained modulation of the overall material’s elastic properties. Biological samples of animal type having a fibrous structure show a stress–strain behavior similar to those determined for both SF/PEDOT:PSS/PVA composites; here, a linearity in the low-stress region indicating elasticity of the system on compression and related deformation and size reduction of pores, followed by a second linear regime at higher applied stress, is found.^42,43^ The elastic modulus by large-area samples of both formulations indicates that 1% PVA-based electrosponges are stiffer (*E* = 485 kPa) due to a higher polymer content than that of the 0.5% PVA biocomposite (*E*= 295 kPa) for the same sample volume. Values of the elastic modulus for the 1% PVA biocomposite are a bit higher than those found using the indenter, also because in this case, the compression may be better distributed over a larger portion of area on the (metallized) cylinder surface. Figure 4C reports the resistivity (ρ) measurements on compression. Accordingly, the SF/PEDOT:PSS/PVA 1% conducting composite shows a far marked decrease of ρ in the low-stress region; hence, such formulation is best suited to finely sense pressure stimuli up to 100 kPa.

It is worth noting that the nominal resistivity values assessed in Figure 4C are likely an underestimation of the actual ones, as the intercalation of metal clusters is expected for a porous material. The same measurement for nonmetallized devices offers resistivity values higher by some orders of magnitude, which are probably an overestimation of the actual value due to bad electrical contacts at the metal/biocomposite interface. Anyway, normalized resistivity vs stress curves for metallized and nonmetallized samples (Figure 4D) show a similar behavior in the analyzed range of applied stresses, with all of them showing variability of resistivity values over many orders of magnitude, up to applied stresses in the order of hundreds of kPa. In Figure 4C,D, it can also be observed that the electrosponge structure collapses due to the compressive structural failure stress, taking place when the applied stress reaches or exceeds the material compressive strength. Due to the highly porous structure of the sponge, the compression may have a different weight on the local response of the composite, as determined by the spatial distribution of plastic trabeculae in the region under stress. Hence, a failure-recovery is simply a result of a local collapse and break of fibers, followed by a redistribution of the conducting rubber-like structure allowing to retain the conductivity. This is argued from the stress–strain measurements (blue curve in Figure 4A) that show a trace of multiple collapse-recovery fingerprints corresponding to a pressure stress of 10^3^ KPa, while the operando resistivity vs applied stress measurements evidence signs of mechanical failure at a lower stress value (10^2^ KPa). Tests conducted on soft materials to check their mechano-structural properties, if based on electrical characterization techniques, can be highly sensitive in probing local effects.^44−46^ In this case, the recovery of the mechanical properties when the structure experiences high compressive stress, approaching the material compressive strength, is hence unfavorably affected. Although the conductivity is somehow preserved, it conversely worsens while approaching the structural failure for the composite, and the normalized resistivity vs stress curves (Figure 4C) better highlight the stress-induced conductivity changes.

### Morphological Characterization of SF and SF/PEDOT:PSS/PVA 3D Structures via μ-CT

A morphological analysis by the μ-CT technique has been carried out on bare SF samples obtained from 2.5% and 5% w/v SF solutions and on 5% w/v SF samples with PEDOT:PSS and both 0.5% and 1% PVA contents. Such analysis aims to both validate and better frame all the features evidenced by mechanical tests in the context of pressure sensing.

Micrographs showing slices of bare SF samples (reported in Figure S3) clearly highlight the greatly porous structure induced by the sequential combination of foaming and fast freezing. In particular, an increased porosity of 94% for the 2.5% bare SF sample is found. The denser porous sample obtained from 5% w/v fibroin solution indeed shows a porosity of 82%. Meanwhile, the mean value of pore wall thickness is preserved (around 30 μm), and the densest samples are characterized by a more homogeneous distribution of wall thickness and pore size, as evidenced by the lower standard deviations of the mean wall thickness and mean wall spacing than those assessed for the 2.5% w/v SF sample. Table S1 reports all the parameters calculated from μ-CT data.

**Figure 5:**
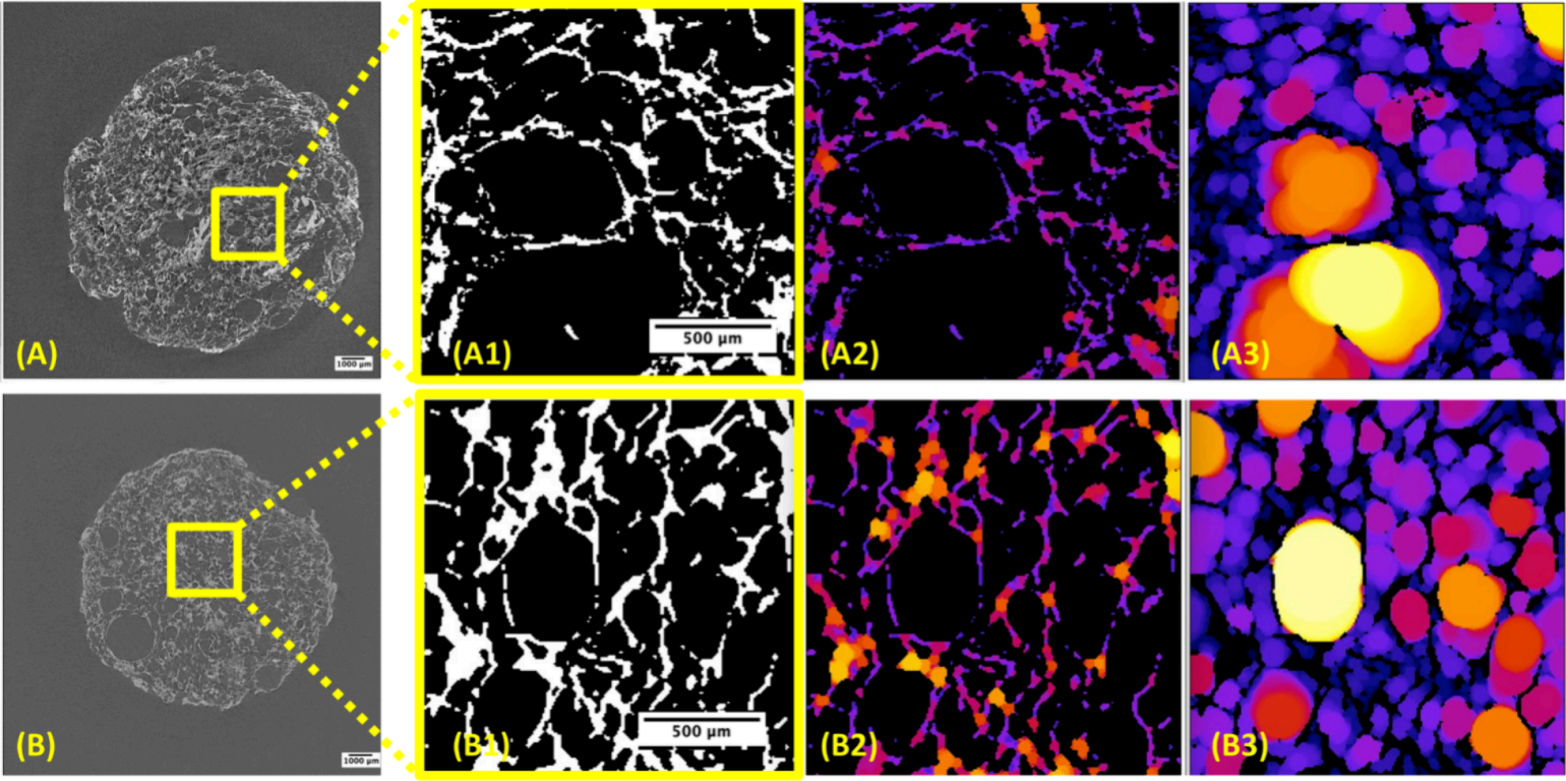
(A) μ-CT image of the SF/PEDOT:PSS/PVA 0.5% v/v foamed sample with magnification of the segmented composite structure (A1) and color maps evidencing the pore walls (A2) and wall spacing (A3) in the foamed/fast-frozen SF/PEDOT:PSS/PVA composite; (B) μ-CT image of the SF/PEDOT:PSS/PVA 1% w/v foamed sample with magnification of the segmented structure (B1) and color maps highlighting the pore walls (B2) and wall spacing (B3).

Micrographs from the μ-CT analysis of SF/PEDOT:PSS/PVA composites are shown in Figure 5.

Having a look at all the morphological parameters extracted from μ-CT data analysis and reported in Table 1, it emerges that the addition of the conducting PEDOT:PSS polymer determines a reduced porosity with respect to that of the polymer-free composite due to the enhancement of both solid volume fraction and pore walls thickness. The lowest standard deviations for the mean values of wall thickness and spacing also indicate an improvement of homogeneity in the distribution of pore dimensions. Nevertheless, the morpho-structure of the as-characterized composites is not dissimilar from that of the PEDOT:PSS-free composite; hence, the further functionality owed to the addition of the conducting polymer into the SF/PVA composites influences but does not strongly alter its elasticity, as also noticed while discussing the stress–strain measurements of Figure 4A. Indeed, the growing trend of the mean wall thickness values from bare SF samples and 0.5% PVA-based SF/PEDOT:PSS/PVA composites (which are both around 30 μm) to 1% PVA-based SF/PEDOT:PSS/PVA (showing thicker walls of 38 μm) explains the origin of the enhanced robustness showed by the latter composite during stress–strain tests.

**Table 1:** Morphological Parameters of the Selected Samples SF/PEDOT:PSS/PVA with PVA Contents of 0.5% w/v and 1% w/v

sample	solid volume (μm^3^)	porosity (%)	mean wall thickness (μm)	max wall thickness (μm)	mean wall spacing (μm)	max wall spacing (μm)
SF (5% w/v)/PEDOT:PSS/PVA (0.5% w/v)	8.6 × 10^8^	79	31(±11)^a^	107	107(±74)^a^	391
SF (5% w/v)/PEDOT:PSS/PVA 1% w/v	1.3 × 10^9^	69	38(±13)^a^	95	105(±63)^a^	403

a Standard deviation.

### Analysis of the Electrical Response by Pressure Sensors Based on SF/PEDOT:PSS/PVA Electrosponges

**Figure 6:**
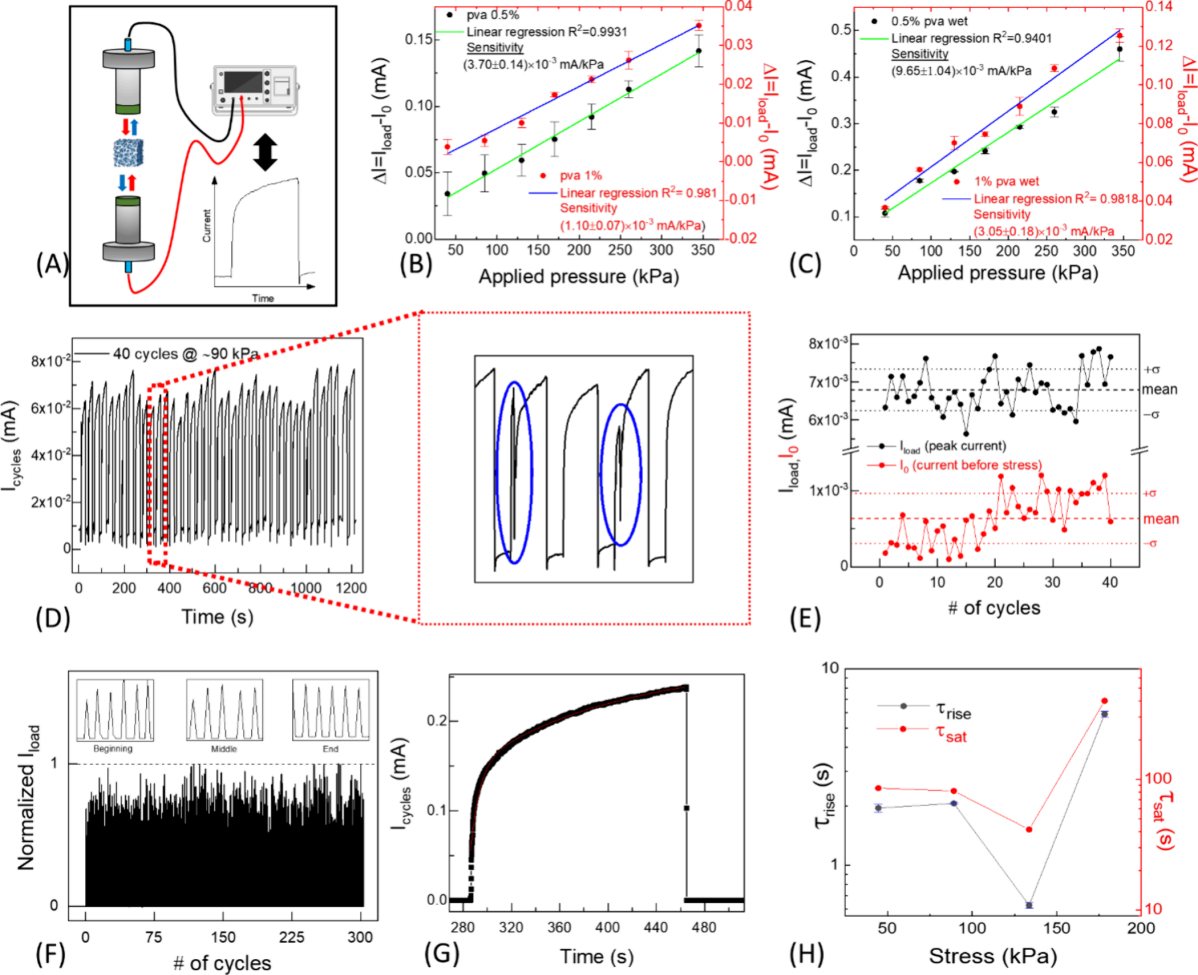
(A) Schematics of the homemade system for pressure sensor testing. (B) Current variation, Δ*I*, as a function of the applied stress recorded in ambient humidity conditions (30% RH) for 0.5% (black symbols) and 1% (red symbols) PVA-based electrosponges and related linear regressions (green and blue lines, respectively). (C) Δ*I* vs applied stress recorded in wet conditions for 0.5% (black symbols) and 1% (red symbols) PVA-based electrosponges and related linear regressions (green and blue lines, respectively). (D) Current vs time on repeated application–removal cycles of an applied pressure of about 90 kPa for a total of 40 cycles (20 s of compression, 10 s of weight removal). Output current on compression at some selected cycles evidence a failure-and-recovery behavior, as highlighted in the magnification. (E) *I*_load_ peak values and *I*_0_ minimum current values assessed before stress removal, both extracted from the curves in panel (D) and reported together with their mean values (dashed lines) and related standard deviations (dotted lines). (F) Current vs time on repeated application–removal cycles of an applied pressure of about 85 kPa for a total of 300 cycles (1 s of compression, 2 s of weight removal). (G) Typical current vs time response under a step-like applied stress and related fitting curve from eq 1 (red line). (H) Rise time and saturation time for SF/PEDOT:PSS/PVA 1% v/v undergoing a compression–decompression cycle for applied pressures of 45–355 kPa.

The pressure sensing capability of the SF/PEDOT:PSS/PVA has been tested by using the homemade apparatus illustrated in Figure 6A and described in the Experimental Section.

Accordingly, current vs time curves are recorded at a fixed voltage bias (*V*_bias_ = 0.5 V, applied between the bases of the cylindrical electrosponge) while compressing and decompressing the cylinders on applying and removing increasing pressure stimuli on their bases. The time variation of the electrical current Δ*I*, expressed as the difference between the current value recorded under compression, *I*_load_, and the one denoted as *I*_0_ corresponding to a basal pressure of about 1.5 kPa, is reported in Figure 6B in both cases (0.5% and 1% PVA-based electrosponges). The 0.5% PVA blend shows a current variation of 1 order of magnitude higher than that of 1% PVA, which may be ascribed to a larger porosity and a lower elastic modulus. Consequently, the former sample even shows an enhanced sensitivity of 3.70 (±0.14) × 10^–3^ mA/kPa, while the sensitivity value for the denser blend is lower by a factor 3, i.e., 1.10 (±0.07) × 10^–3^ mA/kPa). However, large error bars, calculated from repeated Δ*I* vs t measurements as the standard error of the mean, are found for the softer blend at 0.5% PVA, an indication of nonefficient shape recovery ascribable to a less resilient character of the pore walls. Our results can be compared to the literature, with limited papers treating SF-based pressure sensors in a similar manner.^29,30^ In particular, He et al.^29^ and Xu et al.^30^ showed SF sensors with similar performances in pressure sensing parameters, but in both cases, they treated the materials with completely different morpho-structures and synthesized with diverse additional components.

The role of PVA as an elasticity enhancer and reinforcing agent for the porous structure of electrosponges is even more evident from a comparison between measurements of Figure 5B and SF/PEDOT:PSS composite (i.e., PVA-free electrosponge, Figure S4A). Despite showing the highest output response, the latter sample suffers from evident collapse of the structure (Figure S4B) and its fragility only may allow disposable applications.

Blends containing PVA have also been tested in wet conditions by dipping the 3D-shaped material into distilled water for 5 min and then letting it to air-dry for 20 min until the excess water is removed. This study is needed because it is known that the solid state structure of fibrillary silk compounds is affected by hydration to the extent that, if wet, such structures tend to soften, collapsing and easily reaching the rupture under given load conditions.^47^

Compression tests have been performed on the samples with 0.5% and 1% PVA (Figure 6C). Results of compression tests show that the current recorded in response to applied pressures falling into the selected window of values (i.e., between 45 and 355 kPa) is higher in the case of wet samples, and this behavior may be related to the presence of water that softens the material fibers, allowing a major compression. Accordingly, higher sensitivities of 9.65 (±1.04) × 10^–3^ mA/kPa for the 0.5% PVA sample and 3.05 (±0.18) × 10^–3^ mA/kPa for the 1% PVA specimen have been found. It is worth noting that, on wetting, the 3D piezoresistive structure retains the original shape of the dry conditions, it does not break following compression steps, and above all, it tends to restore its shape.

Compression–decompression cycles have been applied to a SF/PEDOT:PSS/PVA 1% v/v sample aimed at recording its response on repeated application and removal of pressure stimuli for material stability/sensor durability check (Figure 6D). Although an occasional failure-and-recovery behavior, as evidenced (blue-circled) in the magnification of Figure 6D, takes place due to local breaks followed by pore redeployment that somehow reminds of the self-healing properties of some polymers such as PEDOT:PSS,^48^ cyclic measurements indicate a good preservation of the device modulation capability over a number of 40 cycles herein tested. In this respect, Figure 6E reports *I*_load_ and *I*_0_ values assessed from the curve of panel D, together with their mean values (dashed lines) and related standard deviations (dotted lines). *I*_0_ experiences a slight step-like enhancement of its values after 20 compression cycles, which may be due to a crossover from an initial regime of the 3D porous structure redistribution (and the failure-and-recovery behavior may be a signature of its influence on shape recovery occurring on decompression) to a more stable structure. *I*_load_ is indeed more stable over 40 cycles, indicating that the crossover does not affect it under applied pressure stimuli; contextually, *I*_load_ shows a relative standard deviation  of 10%, lower than that of *I*_0_ (RDS of 52%).

Figure 6F displays the longer pressure stability tests performed on the 1% PVA composite with faster compression–decompression cycles. The composite maintained its performances after the application of faster mechanical stimuli for prolonged time. The device swiftness has been studied by analyzing the current vs time curves of the 1% PVA-based electrosponge, as described in the Experimental Section. In this respect, the dynamics of the electrosponge settlement on step-like applied pressure is described by τ_rise_, which is a measure of the sensor swiftness, and τ_sat_, connected to the time needed for the current to saturate due to a stabilization of the electrosponge microstructure in response to the applied stress. A typical current vs time response under a step-like applied stress is reported in Figure 6G. Figure 6H reports the τ_rise_ and τ_sat_ assessed by fitting the acquired currents (using eq 1) as a function of the applied stress.

Rise times ranging around 2 s and saturation times around 80 s are found at lower loads, both tending to decrease down to 0.62 and 41 s for loads realizing the lower resistivity values of Figure 4C under sample compression, respectively. A slower sensor response, characterized by τ_rise_ ranging around few seconds, is found at loads compatible with the structural failure inferred from the mechanical and morphological analyses (i.e., τ_rise_ = 5.8 s for an applied stress of 355 kPa).

## Conclusions

In conclusion, we have proposed a foaming methodology for assembling SF/PEDOT:PSS/PVA electrosponges by a green, sustainable, and eco-friendly approach, hence responding to the precepts of a circular economy. An application for pressure sensing has been presented as a case study. The combination of SF, PEDOT:PSS, and PVA as an elasticity enhancer has resulted in a biocompatible, biodegradable, and highly conductive SF conducting composite with excellent mechanical resilience. The beneficial role of mechanical foaming plus freeze-drying and O/N annealing in terms of improved homogeneity and promotion of β-sheets, inducing a stable, crystalline 3D structure, has been highlighted by a dedicated characterization of morphostructural and mechanoelectrical properties shown by formulations differing for PVA percentage content. In particular, besides the lower conductivity induced by PVA addition contrasts the gained resilience and enhanced elasticity by the material (i.e., low elastic moduli in the range of tens-hundreds of kPa that have been related to the as-found highly porous 3D structure induced by the foaming/fast freezing method), the SF/PEDOT:PSS/PVA 1% formulation has shown the best compromise between resilience and shape recovery on 40 cyclic measurements, indicating that the related sensors are able to work in a wide range of applied pressures (up to 90 kPa) with the best response and recovery times below 1 s and far below 1 min, respectively, and with a sensitivity under environmental humidity conditions of 1.10 (±0.07) × 10^–3^ mA/kPa (which becomes 3.05 (±0.18) × 10^–3^ mA/kPa for the as-shown stable and reversible operation in wet conditions), in line with those found in the case of hybrid biopolymer/organic/inorganic compounds in the literature. The proposed electrosponges can be produced with a tuned scalability using solution processable approaches for monolithic integration, such as 3D printing methods for rapid prototyping. Considering the synthesis methodology and the components employed, we propose a material with high structural and environmental versatility, offering potential applications across various fields. The biocompatibility of the components paves the way for applications in wearable and implantable pressure sensors for monitoring human motion parameters, as well as in functional tissue engineering, for example, by exploiting electrical field stimulation approaches. On the other hand, the material’s morphological malleability and monolithic nature facilitate the integration in advanced electronics devices and support the incorporation of morphological adaptation with other materials for next-gen robotics and textile electronics.

## Supporting Information Available

Images acquired from stereoscopy showing the partial interaction between SF and PEDOT:PSS; full FTIR spectra comparison; μ-CT analysis of foamed bare SF samples from 2.5% and 5% fibroin solutions; comparison of responses by pressure sensors based on SF/PEDOT:PSS/PVA 1% and 2% with SF/PEDOT:PSS electrosponges, and real images of both samples before and after pressure sensing tests; table reporting morphological parameters assessed from μ-CT analysis of foamed bare SF samples (PDF)

## References

[ref1] Ansermet S.; Otter D.; Craddock R. W.; Dancaster J. L. Cooperative Development of a Piezoresistive Pressure Sensor with Integrated Signal Conditioning for Automotive and Industrial Applications. Sens Actuators A Phys. 1990, 21 (1–3), 79–83. 10.1016/0924-4247(90)85016-W.

[ref2] Soy H.; Toy İ. Design and Implementation of Smart Pressure Sensor for Automotive Applications. Measurement 2021, 176, 109184 10.1016/j.measurement.2021.109184.

[ref3] Almassri A. M.; Wan Hasan W. Z.; Ahmad S. A.; Ishak A. J.; Ghazali A. M.; Talib D. N.; Wada C. Pressure Sensor: State of the Art, Design, and Application for Robotic Hand. J. Sens 2015, 2015, 1–12. 10.1155/2015/846487.

[ref4] Mishra S.; Mohanty S.; Ramadoss A. Functionality of Flexible Pressure Sensors in Cardiovascular Health Monitoring: A Review. ACS Sens 2022, 7 (9), 2495–2520. 10.1021/acssensors.2c00942. 36036627

[ref5] Chai S.; Wu H.; Peng X.; Tan Z.; Cao H.; Wei L.; Mao X.; Zhang Z.; Zhou F.; Zhang Q.; Sun R.; Liu C. Progress in Research and Application of Modified Silk Fibroin Fibers. Adv. Mater. Technol. 2024, 9 (3), 2301659 10.1002/admt.202301659.

[ref6] Burnie L.; Chockalingam N.; Holder A.; Claypole T.; Kilduff L.; Bezodis N. Testing Protocols and Measurement Techniques When Using Pressure Sensors for Sport and Health Applications: A Comparative Review. Foot 2024, 59, 102094 10.1016/j.foot.2024.102094. 38579518

[ref7] Fong D. T.-P.; Chan Y.-Y.; Hong Y.; Yung P. S.-H.; Fung K.-Y.; Chan K.-M. A Three-Pressure-Sensor (3PS) System for Monitoring Ankle Supination Torque during Sport Motions. J. Biomech 2008, 41 (11), 2562–2566. 10.1016/j.jbiomech.2008.05.035. 18617177

[ref8] Guo Y.; Zhong M.; Fang Z.; Wan P.; Yu G. A Wearable Transient Pressure Sensor Made with MXene Nanosheets for Sensitive Broad-Range Human–Machine Interfacing. Nano Lett. 2019, 19 (2), 1143–1150. 10.1021/acs.nanolett.8b04514. 30657695

[ref9] Hong Q.; Liu T.; Guo X.; Yan Z.; Li W.; Liu L.; Wang D.; Hong W.; Qian Z.; Zhang A.; Wang Z.; Li X.; Wang D.; Mai Z.; Zhao Y.; Yan F.; Xing G. 3D Dual-Mode Tactile Sensor with Decoupled Temperature and Pressure Sensing: Toward Biological Skins for Wearable Devices and Smart Robotics. Sens Actuators B Chem. 2024, 404, 135255 10.1016/j.snb.2023.135255.

[ref10] Guo X.; Zhang T.; Wang Z.; Zhang H.; Yan Z.; Li X.; Hong W.; Zhang A.; Qian Z.; Zhang X.; Shu Y.; Wang J.; Hua L.; Hong Q.; Zhao Y. Tactile Corpuscle-Inspired Piezoresistive Sensors Based on (3-Aminopropyl) Triethoxysilane-Enhanced CNPs/Carboxylated MWCNTs/Cellulosic Fiber Composites for Textile Electronics. J. Colloid Interface Sci. 2024, 660, 203–214. 10.1016/j.jcis.2024.01.059. 38244489

[ref11] Cui C.; Fu Q.; Meng L.; Hao S.; Dai R.; Yang J. Recent Progress in Natural Biopolymers Conductive Hydrogels for Flexible Wearable Sensors and Energy Devices: Materials, Structures, and Performance. ACS Appl. Bio Mater. 2021, 4 (1), 85–121. 10.1021/acsabm.0c00807. 35014278

[ref12] Gupta P.; Nayak K. K. Characteristics of Protein-Based Biopolymer and Its Application. Polym. Eng. Sci. 2015, 55 (3), 485–498. 10.1002/pen.23928.

[ref13] Koh L.-D.; Cheng Y.; Teng C.-P.; Khin Y.-W.; Loh X.-J.; Tee S.-Y.; Low M.; Ye E.; Yu H.-D.; Zhang Y.-W.; Han M.-Y. Structures, Mechanical Properties and Applications of Silk Fibroin Materials. Prog. Polym. Sci. 2015, 46, 86–110. 10.1016/j.progpolymsci.2015.02.001.

[ref14] Vepari C.; Kaplan D. L. Silk as a Biomaterial. Prog. Polym. Sci. 2007, 32 (8–9), 991–1007. 10.1016/j.progpolymsci.2007.05.013. 19543442 PMC2699289

[ref15] Rockwood D. N.; Preda R. C.; Yücel T.; Wang X.; Lovett M. L.; Kaplan D. L. Materials Fabrication from Bombyx Mori Silk Fibroin. Nat. Protoc 2011, 6 (10), 1612–1631. 10.1038/nprot.2011.379. 21959241 PMC3808976

[ref16] Reizabal A.; Costa C. M.; Pérez-Álvarez L.; Vilas-Vilela J. L.; Lanceros-Méndez S. Silk Fibroin as Sustainable Advanced Material: Material Properties and Characteristics, Processing, and Applications. Adv. Funct Mater. 2023, 33 (3), 2210764 10.1002/adfm.202210764.

[ref17] De Giorgio G.; Matera B.; Vurro D.; Manfredi E.; Galstyan V.; Tarabella G.; Ghezzi B.; D’Angelo P. Silk Fibroin Materials: Biomedical Applications and Perspectives. Bioengineering 2024, 11 (2), 167 10.3390/bioengineering11020167. 38391652 PMC10886036

[ref18] Nazarov R.; Jin H.-J.; Kaplan D. L. Porous 3-D Scaffolds from Regenerated Silk Fibroin. Biomacromolecules 2004, 5 (3), 718–726. 10.1021/bm034327e. 15132652

[ref19] Maniglio D.; Bonani W.; Migliaresi C.; Motta A. Silk Fibroin Porous Scaffolds by N _2_ O Foaming. J. Biomater Sci. Polym. Ed 2018, 29 (5), 491–506. 10.1080/09205063.2018.1423811. 29297760

[ref20] Wang F.; Liu H.; Li Y.; Li Y.; Ma Q.; Zhang J.; Hu X. Tunable Biodegradable Polylactide–Silk Fibroin Scaffolds Fabricated by a Solvent-Free Pressure-Controllable Foaming Technology. ACS Appl. Bio Mater. 2020, 3 (12), 8795–8807. 10.1021/acsabm.0c01157. 35019555

[ref21] Suamte L.; Tirkey A.; Barman J.; Jayasekhar Babu P. Various Manufacturing Methods and Ideal Properties of Scaffolds for Tissue Engineering Applications. Smart Materials in Manufacturing 2023, 1, 100011 10.1016/j.smmf.2022.100011.

[ref22] Kayser L. V.; Lipomi D. J. Stretchable Conductive Polymers and Composites Based on PEDOT and PEDOT:PSS. Adv. Mater. 2019, 31 (10), 1806133 10.1002/adma.201806133. PMC6401235 30600559

[ref23] Rinaldi G.; Vurro D.; Cicolini M.; Babic J.; Liboà A.; Tarabella G.; D’Angelo P.; Marasso S. L.; Cocuzza M.; Vigna L.; Pirri F. C.; Parmeggiani M. PEDOT:PSS Deposition in OECTs: Inkjet Printing, Aerosol Jet Printing and Spin Coating. Micro and Nano Engineering 2024, 24, 100272 10.1016/j.mne.2024.100272.

[ref24] Yang T.; Yang M.; Xu C.; Yang K.; Su Y.; Ye Y.; Dou L.; Yang Q.; Ke W.; Wang B.; Luo Z. PEDOT:PSS Hydrogels with High Conductivity and Biocompatibility for *in Situ* Cell Sensing. J. Mater. Chem. B 2023, 11 (14), 3226–3235. 10.1039/D3TB00014A. 36960662

[ref25] Escobar A.; Serafin A.; Carvalho M. R.; Culebras M.; Cantarero A.; Beaucamp A.; Reis R. L.; Oliveira J. M.; Collins M. N. Electroconductive Poly(3,4-Ethylenedioxythiophene) (PEDOT) Nanoparticle-Loaded Silk Fibroin Biocomposite Conduits for Peripheral Nerve Regeneration. Adv. Compos Hybrid Mater. 2023, 6 (3), 118 10.1007/s42114-023-00689-2.

[ref26] Bhattacharjee P.; Ahearne M. Fabrication and Biocompatibility of Electroconductive Silk Fibroin/PEDOT: PSS Composites for Corneal Epithelial Regeneration. Polymers (Basel) 2020, 12 (12), 3028 10.3390/polym12123028. 33348815 PMC7766233

[ref27] Chen M.; Liu J.; Hu Y.; Wu Y.; Tang C.-Y.; Ke K.; Yang W. Silk Fibroin-Based Flexible Pressure Sensors: Processing and Application. Materials Futures 2024, 3 (3), 032501 10.1088/2752-5724/ad5f48.

[ref28] Zhang S.; Zhou Z.; Zhong J.; Shi Z.; Mao Y.; Tao T. H. Body-Integrated, Enzyme-Triggered Degradable, Silk-Based Mechanical Sensors for Customized Health/Fitness Monitoring and In Situ Treatment. Advanced Science 2020, 7 (13), 1903802 10.1002/advs.201903802. 32670755 PMC7341100

[ref29] He F.; You X.; Gong H.; Yang Y.; Bai T.; Wang W.; Guo W.; Liu X.; Ye M. Stretchable, Biocompatible, and Multifunctional Silk Fibroin-Based Hydrogels toward Wearable Strain/Pressure Sensors and Triboelectric Nanogenerators. ACS Appl. Mater. Interfaces 2020, 12 (5), 6442–6450. 10.1021/acsami.9b19721. 31935061

[ref30] Xu M.; Cai H.; Liu Z.; Chen F.; Wang Y.; Dai F.; Li Z. Skin-Friendly Corrugated Multilayer Microspherical Sensor Fabricated with Silk Fibroin, Poly (Lactic-Co-Glycolic Acid), Polyaniline, and Kappa-Carrageenan for Wide Range Pressure Detection. Int. J. Biol. Macromol. 2022, 194, 755–762. 10.1016/j.ijbiomac.2021.11.122. 34838861

[ref31] Li S.; Liu A.; Qiu W.; Wang Y.; Liu G.; Liu J.; Shi Y.; Li Y.; Li J.; Cai W.; Park C.; Ye M.; Guo W. An All-Protein Multisensory Highly Bionic Skin. ACS Nano 2024, 18 (5), 4579–4589. 10.1021/acsnano.3c12525. 38258755

[ref32] Turco G.; Marsich E.; Bellomo F.; Semeraro S.; Donati I.; Brun F.; Grandolfo M.; Accardo A.; Paoletti S. Alginate/Hydroxyapatite Biocomposite For Bone Ingrowth: A Trabecular Structure With High And Isotropic Connectivity. Biomacromolecules 2009, 10 (6), 1575–1583. 10.1021/bm900154b. 19348419

[ref33] Preziosi V.; Barra M.; Perazzo A.; Tarabella G.; Romeo A.; Marasso S. L.; D’Angelo P.; Iannotta S.; Cassinese A.; Guido S. Monitoring Emulsion Microstructure by Using Organic Electrochemical Transistors. J. Mater. Chem. C Mater. 2017, 5 (8), 2056–2065. 10.1039/C6TC05149A.

[ref34] Yucel T.; Cebe P.; Kaplan D. L. Vortex-Induced Injectable Silk Fibroin Hydrogels. Biophys. J. 2009, 97 (7), 2044–2050. 10.1016/j.bpj.2009.07.028. 19804736 PMC2756352

[ref35] Li X.; Fan Q.; Zhang Q.; Yan S.; You R. Freezing-Induced Silk I Crystallization of Silk Fibroin. CrystEngComm 2020, 22 (22), 3884–3890. 10.1039/D0CE00360C.

[ref36] Tamada Y. New Process to Form a Silk Fibroin Porous 3-D Structure. Biomacromolecules 2005, 6 (6), 3100–3106. 10.1021/bm050431f. 16283733

[ref37] Khosravimelal S.; Chizari M.; Farhadihosseinabadi B.; Moosazadeh Moghaddam M.; Gholipourmalekabadi M. Fabrication and Characterization of an Antibacterial Chitosan/Silk Fibroin Electrospun Nanofiber Loaded with a Cationic Peptide for Wound-Dressing Application. J. Mater. Sci. Mater. Med. 2021, 32 (9), 114 10.1007/s10856-021-06542-6. 34455501 PMC8403119

[ref38] Libera V.; Malaspina R.; Bittolo Bon S.; Cardinali M. A.; Chiesa I.; De Maria C.; Paciaroni A.; Petrillo C.; Comez L.; Sassi P.; Valentini L. Conformational Transitions in Redissolved Silk Fibroin Films and Application for Printable Self-Powered Multistate Resistive Memory Biomaterials. RSC Adv. 2024, 14 (31), 22393–22402. 10.1039/D4RA02830A. 39010927 PMC11248567

[ref39] Rajesha Shetty G.; Lakshmeesha Rao B. Preparation and Characterization of Silk Fibroin-Polyvinyl Alcohol (PVA) Blend Films for Food Packaging Materials. Mater. Today Proc. 2022, 55, 194–200. 10.1016/j.matpr.2022.02.034.

[ref40] Kim N.; Lienemann S.; Petsagkourakis I.; Alemu Mengistie D.; Kee S.; Ederth T.; Gueskine V.; Leclère P.; Lazzaroni R.; Crispin X.; Tybrandt K. Elastic Conducting Polymer Composites in Thermoelectric Modules. Nat. Commun. 2020, 11 (1), 1424 10.1038/s41467-020-15135-w. 32188853 PMC7080746

[ref41] Adekoya G. J.; Adekoya O. C.; Sadiku E. R.; Hamam Y.; Ray S. S. Effect of Borophene and Graphene on the Elastic Modulus of PEDOT:PSS Film—A Finite Element Study. Condens Matter 2022, 7 (1), 22 10.3390/condmat7010022.

[ref42] Duenwald-Kuehl S.; Kondratko J.; Lakes R. S.; Vanderby R. Damage Mechanics of Porcine Flexor Tendon: Mechanical Evaluation and Modeling. Ann. Biomed Eng. 2012, 40 (8), 1692–1707. 10.1007/s10439-012-0538-z. 22399329

[ref43] Jang S.; Vanderpool R. R.; Avazmohammadi R.; Lapshin E.; Bachman T. N.; Sacks M.; Simon M. A. Biomechanical and Hemodynamic Measures of Right Ventricular Diastolic Function: Translating Tissue Biomechanics to Clinical Relevance. J. Am. Heart Assoc 2017, 6 (9), e006084 10.1161/JAHA.117.006084. 28899895 PMC5634275

[ref44] Krajči J.; Špitálský Z.; Chodák I. Relationship between Conductivity and Stress–Strain Curve of Electroconductive Composite with SBR or Polycaprolactone Matrices. Eur. Polym. J. 2014, 55, 135–143. 10.1016/j.eurpolymj.2014.03.013.

[ref45] Cai W.; Jomdecha C.; Zhao Y.; Wang L.; Xie S.; Chen Z. Quantitative Evaluation of Electrical Conductivity inside Stress Corrosion Crack with Electromagnetic NDE Methods. Philosophical Transactions of the Royal Society A: Mathematical, Physical and Engineering Sciences 2020, 378 (2182), 20190589 10.1098/rsta.2019.0589. 32921234

[ref46] Ali M. S.; Parvin R. Investigation of Pressure Effect on Structural, Mechanical, Electrical and Optical Properties of AlAs for Optoelectronic Applications. Physica B Condens Matter 2024, 673, 415491 10.1016/j.physb.2023.415491.

[ref47] Chen J.; Yin Z.; Tan G.; Xing T.; Kundu S. C.; Lu S. Research on Silk Fibroin Composite Materials for Wet Environment Applications. J. Mech Behav Biomed Mater. 2024, 160, 106777 10.1016/j.jmbbm.2024.106777. 39418745

[ref48] Zhang S.; Cicoira F. Water-Enabled Healing of Conducting Polymer Films. Adv. Mater. 2017, 29 (40), 1703098 10.1002/adma.201703098. 28846168

