# Supplementary material for: Breakthrough Assembly of a Silk Fibroin Composite for Application in Resistive Pressure Sensing
Source: ACS Appl Polym Mater. 2025 Apr 10;7(8):5013–24. doi: 10.1021/acsapm.5c00242 (PMC12080453; doi:10.1021/acsapm.5c00242)
Supplement: Supplementary file 1 — ap5c00242_si_001.pdf [file ap5c00242_si_001.pdf]

Supporting Information

Breakthrough Assembling of a Silk Fibroin Composite for Application in Resistive Pressure Sensing

Giuseppe De Giorgio[1,‡], Valentina Vit[1,‡], Davide Vurro[1], Benedetta Guagnini[2], Bianca Zumbo[2]*

Nicola Coppedè[1], Gianluca Turco[2], Giuseppe Tarabella[1,], Pasquale D'Angelo[1]*

[1]Institute of Materials for Electronics and Magnetism (IMEM), National Research Council (CNR), P.co Area delle Scienze 37/A, 43124, Parma (Italy)

[2] Department of Medicine, Surgery and Health Sciences, University of Trieste, Piazza dell'Ospitale 1, 34129, Trieste, Italy

Corresponding authors mail addresses:

Giuseppe De Giorgio: giuseppedegiorgio@cnr.it

Giuseppe Tarabella: giuseppe.tarabella@cnr.it

Contents:

- *S1 Images of the SF/PEDOT:PSS composite from stereoscopy*
- *S2 FTIR spectra of SF, SF/PEDOT:PSS and SF/PEDOT:PSS/PVA 1% v/v*
- *S3 μ-CT analysis of SF 3D shaped samples from aqueous fibroin 2.5% and 5 % w/v*
- *S4 Comparison between ΔI vs. pressure response of SF/PEDOT:PSS and related curves assessed for SF/PEDOT:PSS/PVA composites*
- *Table S1 Morphological parameters of SF2.5% w/v and 5% w/v samples from μ-CT analysis*

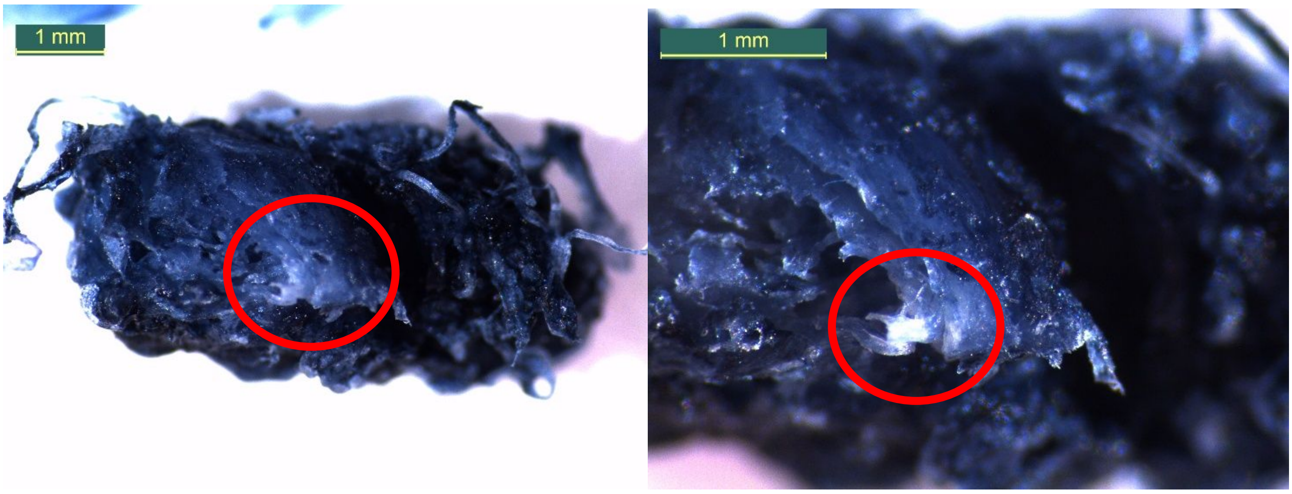

Figure S1. *Images acquired from stereoscopy on the fibrous dry material, showing the partial interaction between SF and PEDOT:PSS blend occuring from the primary interaction of the two components. It is clearly visible a whitish region (red circled) suggesting the presence of fibroin that has not fully interacted with the PEDOT:PSS conducting polymer.*

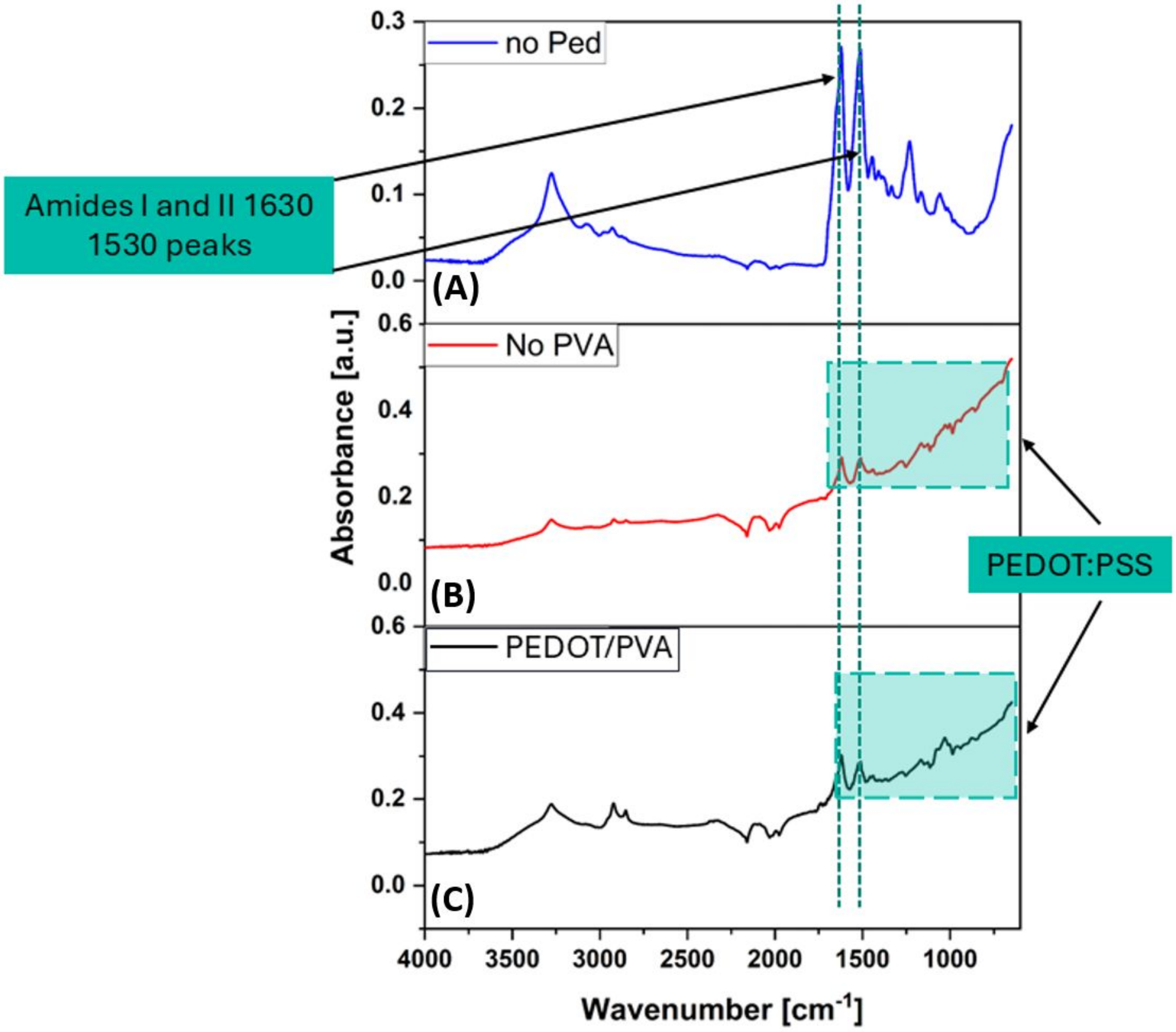

S2. *FTIR spectra of (A) SF, (B) SF/PEDOT:PSS and (C) SF/PEDOT:PSS/PVA 1% v/v; spectral parts corresponding to PEDOT:PSS and Amides peaks are highlighted.*

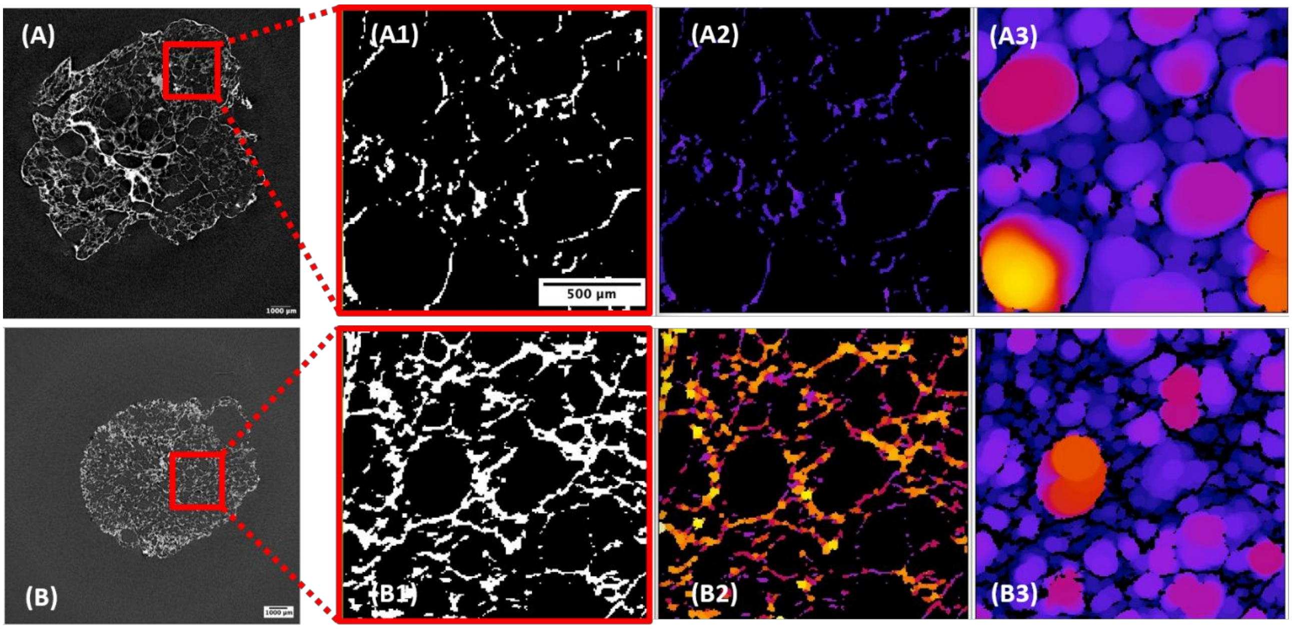

Figure S3. *(A) µ-CT image of SF 2.5% w/v foamed sample with magnification of the segmented composite structure (A1) and color maps evidencing the pore walls (A2) and walls spacing (A3) in the foamed/fast-frozen SF 3D-shaped material; (B) µ-CT image of SF 5% w/v sample with magnification of segmented structure (B1) and color maps highlighting the pore walls (B2) and walls spacing (B3)*

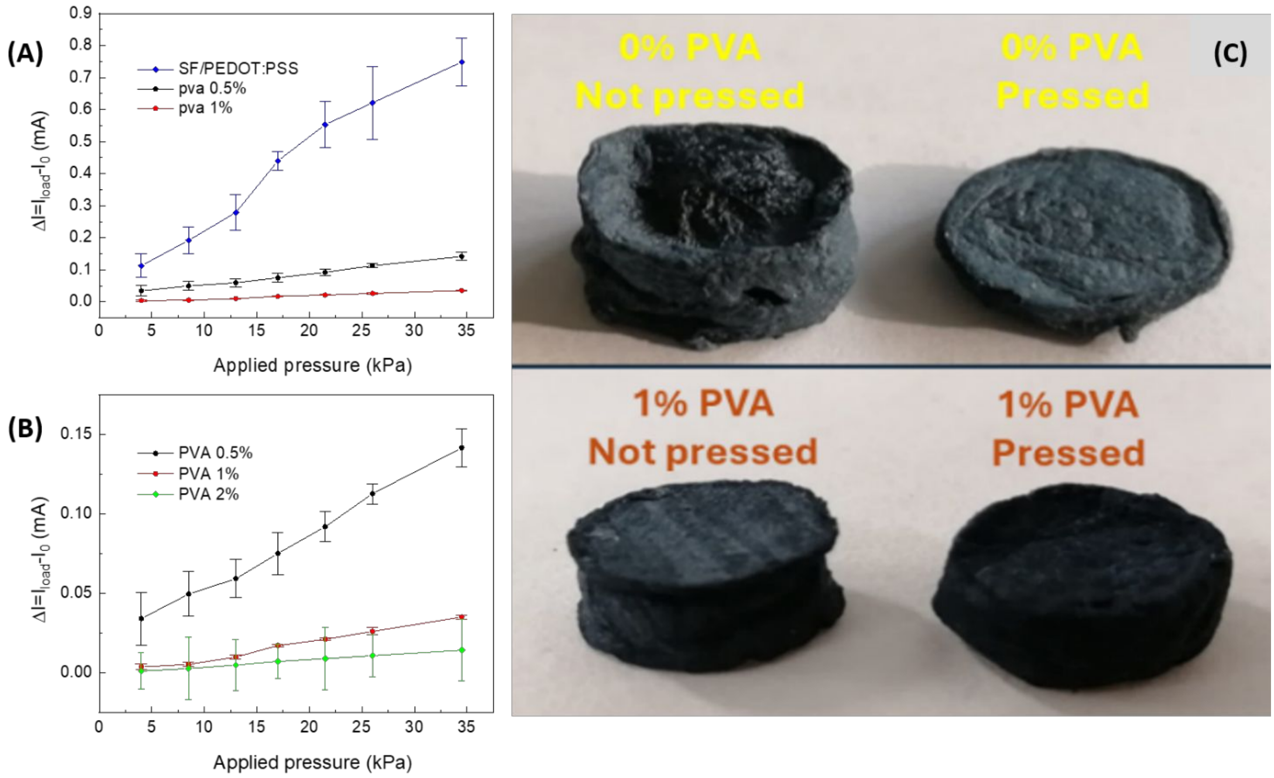

Figure S4. *(A) ΔI vs. applied pressure for SF/PEDOT:PSS, SF/PEDOT:PSS/PVA 0.5% and SF/PEDOT:PSS/PVA 1% (error bars are the standard error of the mean for repeated measurements); (B) images of SF/PEDOT:PSS (upper panel) and SF/PEDOT:PSS/PVA 1% (lower panel) before (left) and after (right) compression, addressing the role of PVA as elasticity enhancer; (C) ΔI vs. applied pressure for SF/PEDOT:PSS/PVA 0.5%, SF/PEDOT:PSS/PVA 1% and SF/PEDOT:PSS/PVA 2% (error bars are the standard error of the mean for repeated measurements) .*

Sample	Solid volume (μm^3)	Porosity (%)	Mean wall thickeness (μm)	Max wall thickness (μm)	Mean wall spacing (μm)	Max wall spacing (μm)
SF 2.5% w/v	2.4×10^8	94	29(±20)*	141	325(±251)*	963
SF 5% w/v	7.3×10^8	82	28(±7)*	58	126(±83)*	499

(*standard deviation)

***Table S1**: Morphological parameters of the selected samples SF2.5% w/v and 5% w/v (*standard deviations).*
